# A Lifespan Observation of a Novel Mouse Model: *In Vivo* Evidence Supports Aβ Oligomer Hypothesis

**DOI:** 10.1371/journal.pone.0085885

**Published:** 2014-01-21

**Authors:** Yichi Zhang, Lu Lu, Jianping Jia, Longfei Jia, Changiz Geula, Jinjing Pei, Zhiqing Xu, Wei Qin, Ruiqin Liu, Dan Li, Na Pan

**Affiliations:** 1 Department of Neurology, Xuan Wu Hospital, Capital Medical University, Beijing, China; 2 Beijing Key Laboratory of Geriatric Cognitive Disorders, Beijing, China; 3 Key Neurodegenerative Laboratory of Ministry of Education of the People's Republic of China, Beijing, China; 4 Department of Neurology, Beijing Tong Ren Hospital, Capital Medical University, Beijing, China; 5 Department of Neurobiology, Karolinska Institutet, Stockholm, Sweden; 6 Laboratory for Cognitive and Molecular Morphometry, Cognitive Neurology and Alzheimer's Disease Center, Northwestern University, Chicago, Illinois, United States of America; 7 Department of Neurobiology, Capital Medical University, Beijing, China; Centre Hospitalier de l'Université Laval, Canada

## Abstract

Transgenic mouse models are powerful tools in exploring the mechanisms of AD. Most current transgenic models of AD mimic the memory impairment and the main pathologic features, among which the formation of beta-amyloid (Aβ) plaques is considered a dominant pathologic event. Recently, Aβ oligomers have been identified as more neurotoxic than Aβ plaques. However, no ideal transgenic mouse model directly support Aβ oligomers as a neurotoxic species due to the puzzling effects of amyloid plaques in the more widely-used models. Here, we constructed a single-mutant transgenic (Tg) model harboring the PS1_V97L_ mutation and used Non-Tg littermates as a control group. Employing the Morris water maze, electrophysiology, immunohistochemistry, biochemistry, and electron microscopy, we investigated behavioral changes and pathology progression in our single-mutant transgenic model. We discovered the pathological alteration of intraneuronal accumulation of Aβ oligomers without Aβ plaques in the PS1_V97L_-Tg mouse model, which might be the result of PS1 gene mutation. Following Aβ oligomers, we detected synaptic alteration, tau hyperphosphorylation and glial activation. This model supports an initial role for Aβ oligomers in the onset of AD and suggests that Aβ plaques may not be the only prerequisite. This model provides a useful tool for studying the role of Aβ oligomers in AD pathogenesis.

## Introduction

Alzheimer's disease (AD) is a progressive neurodegenerative disorder and the most common cause of dementia in the aging population. Researchers found that approximately half of familial AD (FAD) can be attributed to certain gene variations in amyloid precursor protein (APP), presenilin-1 (PS1) and presenilin-2 (PS2). This observation provides a basis for establishing transgenic animal models to explore the possible causal effects of these mutations on the classic AD pathology: extracellular β-amyloid (Aβ) plaques, intraneuronal neurofibrillary tangles (NFTs), synaptic loss and neuronal degeneration. The first successful AD transgenic mouse model with a single mutation was the APP V717F line with apparent Aβ plaques, which showed neither NFTs nor neuronal loss, except for a small degree of synaptic loss [1]. Similar findings were described in Tg2576 and APP23 mice [2], [3]. Meanwhile, research on PS1 transgenic mice presented several challenges in the early study. Almost all attempts failed to produce any of the typical AD-like pathology in PS1 transgenic mice, except for high expression of Aβ in the brain [4]–[6]. While APP or PS1 transgenic mice failed to develop NFTs and neuronal loss, transgenic mice with microtubule-associated protein tau (MAPT) gene such as: JNPL3 mice [7], Htau mice [8] and rTg4510 mice [9], were found to exhibit these two pathologic features. However, in clinical practice, MAPT mutation is usually associated with tauopathy and frontotemporal dementia rather than AD.

It was later observed that expression of PS1 mutations in APP-Tg mice could accelerate Aβ aggregation into plaques compared with single mutant APP-Tg mice [10]. This finding encouraged the creation of other multi-transgenic mice in pursuit of animal models with more robust pathologic features. For example, the triple transgenic model (3×Tg-AD) exhibited both Aβ plaques and NFTs, while 5×Tg-AD mice displayed intraneuronal accumulation of Aβ from as early as approximately 1.5 months old, followed by plaques and neuronal loss [11], [12]. However, the clinical practice literature reports that in most FAD patients, only one mutation is present, which is a marked contrast with the multi-transgenic models and may be problematic, even though the multi-transgenic models develop more typical AD pathology [13]. Additionally, the multi-transgenic mice are more complex and expensive on establishing, mouse line maintaining and genotype identifying. Therefore, an ideal AD transgenic animal model should carry only one gene mutation and exhibit both the cognition deficits and the major pathologic features of AD.

Our group was the first to report a single missense mutation Val97Leu (V97L) of PS1 in a Chinese pedigree suffering from early onset AD [14], and we generated a transgenic mouse line bearing the PS1 V97L mutation (PS1_V97L_-Tg) [15]. In the previous study, Wang et al., detected abnormal behavioral change in PS1_V97L_-Tg mice and correlated this change with abnormal tau hyperphosphorylation [15]. However, we know that PS1 is essential for APP proteolysis. Mutation in PS1 gene usually causes changes in Aβ production; it can either increase Aβ level or changes the proportion of Aβ42/Aβ40 or toxic Aβ species. Abnormal tau production is not the only ‘original sin’ in AD pathogenesis cascade attributed to PS1 V97L mutation and some factors other than pathological tau might play a more direct role. In the present study, although there was no plaque, we detected intracellular toxic Aβ species, Aβ oligomers in PS1_V97L_-Tg mice. To elucidate the potential correlated relationship between Aβ oligomers and the other pathological changes, we conducted a comprehensive investigation of pathology and cognitive dysfunction in PS1_V97L_-Tg mice throughout their lifespan.

## Materials and Methods

### Ethics Statement

All animal experiments were approved by the Ethics Committee of Capital Medical University (2010-X-098), and every effort was made to minimize the number and suffering of animals.

### Animals

PS1_V97L_-Tg mice expressing the human PS1 gene with the V97L mutation were generated as previously described [15]. PS1_V97L_-Tg mouse lines were maintained by crossing heterozygous transgenic mice with wild type C57BL/6J animals. Thus, the PS1_V97L_-Tg mice used were heterozygous for the V97L mutant transgene. Mice were screened by polymerase chain reaction (PCR) to determine their genotypes as previously described [15]. Non-Tg littermates served as a negative control and Tg2576 mice, a well-known model of AD that exhibits massive plaques, served as a positive control. The latter were purchased from the Institute of Laboratory Animal Sciences, Peking Union Medical College. For the quantitative experiments, the control and PS1_V97L_-Tg groups each contained 6 mice; otherwise, unless specifically indicated, each group contained 3 animals for replication.

### Behavioral tests

Spatial memory in mice was assessed at 6 and 9 months of age using Morris Water Maze (MWM), essentially as described in Nature Protocol [16], the conditions of which were not identical to Wang et al. 's article [15] but are also well accepted. Mice were housed in 12 h: 12 h light-dark cycle (lights on at 18:00, and off at 06:00) to ensure that the tests were carried out within their active period. The mice were divided into two groups: the Non-Tg group and the PS1_V97L_-Tg group; for 5 consecutive days, both groups were trained to find a platform hidden below the water surface in a pool with a diameter of 100 cm. MWM was carried out by coworkers who were unaware of the genotypes of mice. Training consisted of four trials per day with an intertrial interval of 30 sec. On the 6th day, the retention of the trained spatial memory was assessed using a probe trial in which mice performed a 30 sec free-searching of the pool with the platform removed. The frequency of crossing the “platform position” was recorded by DNS-2 type Morris Water Maze testing set with an online video tracking system (camera, TOTA-450III, Japan).

### Electrophysiology

Acute coronal hippocampal slices (350 µm thick) were prepared as previously described [17], [18]. Briefly, slices were cut on a Vibratome in ice-cold artificial cerebrospinal fluid (aCSF) that was bubbled with a mixture of 95% O_2_ and 5% CO_2_. The aCSF contained 126 mM NaCl, 2.5 mM KCl, 1.25 mM NaH_2_PO_4_, 26 mM NaHCO_3_, 10 mM D-glucose, 2 mM MgSO_4_ and 2 mM CaCl_2_ and had a pH of 7.4. Slices were transferred to a submerged chamber that was continuously perfused with oxygen-enriched aCSF at 29°C, and fEPSPs elicited by SC fiber stimulation were recorded from the CA1 stratum radiatum. Baseline recordings were made for a minimum of 10 min to ensure stabilization of responses before experiments were initiated. For PPF, the stimulation interval was 20 ms. A tetanic stimulation (100 Hz, 1 sec) was used to induce LTP. Recordings were made using an EPC 10 amplifier (HECA, Germany) and Igor pro 6.2 software (WaveMetrics, USA) was used for the data analysis.

### Antibodies

All antibodies applied were commercially purchased: mouse monoclonal antibody to β-Amyloid, 17–24 (4G8) (SIG-39245, Covance, CA, USA); rabbit polyclonal antibody A11 to Aβ oligomers (Error! Hyperlink reference not valid., Invitrogen, CA, USA); mouse monoclonal antibody to pathological tau, AT-8(pSer202/Thr205) (MN1020, Thermo Scientific, IL, USA); rabbit antibody to synaptophysin, a presynaptic marker (Sigma Aldrich, USA); rabbit polyclonal antibody to Iba-1, a marker of activated microglia (019-19741, Wako Chemicals, VA, USA); NeuN, a marker of mature neurons (Millipore, MA, USA); and β-actin (sc-81178, Santa Cruz, USA).

### Immunohistochemistry

Mice were anesthetized with pentobarbital and transcardially perfused with 4% paraformaldehyde in 0.1 M PBS (pH 7.4). Mouse brains were postfixed in 4% paraformaldehyde overnight, embedded in paraffin, coronally sectioned at a thickness of 5 µm, and deparaffinized with xylene and ethanol. The sections selected were relative to the median sagittal line 2.055 mm, 2.88 mm and 7.05 mm posterior to the Bregma point [19]. Brain regions, including the thalamus, entorhinal cortex, brainstem and cerebellum, were observed. After washing with 0.01 M PBS (pH 7.4), sections were treated with 3% H_2_O_2_ for 10 min to inactivate endogenous peroxidases and then stained with a horseradish peroxidase (HRP) labeled second antibody (otherwise this step could be skipped). To expose epitopes, sections stained with A11, 4G8 and NeuN, were pretreated with 0.01 M citric buffer (pH 6.0) in a microwave for 15 min. As to brain slices stained with synaptophysin, AT-8, Iba-1, GFAP, incubate the slices for 10 min with PBS containing 0.3% Triton X-100. Then, sections were blocked with 10% goat serum in PBS for 30 min at 37°C. Sections were incubated with one of the following primary antibodies: A11 (1∶200), 4G8 (1∶500), AT-8 (1∶30), GFAP (1∶500) Iba-1 (1∶500), andNeuN (1∶500) at 4°C for 24 h. Primary antibody incubation was followed by incubation with an HRP-labeled second antibody, and then, staining was visualized with substrate DAB using the GTVision™ III Detection System kit (Gene Tech Company Limited, China). Synaptophysin was stained using a specific primary antibody (1∶200) followed by Alexa Fluor® 488 Goat Anti-Rabbit IgG (H+L) (1∶400) (Invitrogen, USA). Thioflavin-S (T1892-25G, Sigma-Aldrich, USA) staining, which recognizes the β-pleated sheet abnormal protein conformation in neurofibrillary tangles and mature compact amyloid plaques, was performed as described previously [20]. A Nikon 80I system consisting of a microscope and a digital camera was used to examine and photograph the sections.

### Biochemistry

Western blotting was used to estimate total levels of target proteins (synaptophysin, AT-8, Iba-1, GFAP, β-actin) in cortex and hippocampus of mice brain at 9 and 12 months of age. Mice were immediately decapitated without anesthesia and brain tissue from the regions of interest was rapidly dissected, frozen in liquid nitrogen for 30 min and stored at −80°C. Separately, frozen cortices and hippocampi were thawed and then mixed gently in a mortar with a few strokes of a pestle in 5 wet weight volumes of ice-cold Tris-buffered saline (TBS) containing a Complete Mini Protease Inhibitor Cocktail Tablet (Roche, Germany) and a Phosphatase Inhibitor Cocktail Tablet (Roche, Germany), at a final dilution of 1∶100. The homogenates were centrifuged at 100,000×*g* for 1 h at 4°C, and the supernatants were harvested. Protein concentrations were determined using a BCA Protein Assay Kit (Thermo Scientific, USA). For each sample, 150 µg of total protein were separated on 10% SDS-PAGE gel and transferred onto a nitrocellulose membrane. The membranes were blocked in a solution of 5% fat-free milk for 30 min at 20°C and incubated overnight at 4°C with one of the following primary antibodies: AT-8 (1∶80), synaptophysin (1∶200), Iba-1 (1∶400), GFAP (1∶400) or β-actin (1∶1000). Primary antibody incubation was followed by incubation at 37°C for 1 h with the HRP-labeled secondary antibody and finally by visualization using enhanced chemiluminescence reagents (Beyotime Institute of Biotechnology, China). The membranes were scanned (Alpha Innotech, USA), and optical densities were determined using ImageJ software (v1.46; National Institutes of Health). ELISA of Aβ40 and Aβ42 was performed using commercial kits (Mouse/Rat Amyloid β 1-40 Assay Kit and Mouse/Rat Amyloid β 1-42 Assay Kit) purchased from IBL, by following the manufacturer's instructions.

### Electron microscopy

Twelve-month-old mice were anesthetized with pentobarbital and transcardially perfused with a fixative composed of 2.5% glutaraldehyde and 4% paraformaldehyde in 0.1 M PBS (pH 7.4). The CA3 region was dissected into 1 mm^3^ samples and fixed for 2 h in 3% glutaraldehyde and then rinsed in 0.1 M PBS (pH 7.4). Samples were postfixed for 2 h in 1% osmium tetroxide. Tissue was dehydrated in graded solutions of ethanol from 50% to 100% (each for 10 min), and embedded in EMBed 812. Ultrathin sections (70 nm) were cut using an ultramicrotome (Leica, Germany). Sections were counterstained with uranyl acetate, followed by lead nitrate and observed with a type 2100 transmission electron microscope (JEOL, Japan). Ultrastructural quantification of the synapses was performed using electron microscopy as described previously [21]. Synapses were identified by the presence of a postsynaptic density in association with the postsynaptic element and synaptic vesicles in a presynaptic terminal. Counting of the synapses was performed by a coworker who was unaware of the origin of the images, and the difference in synapse numbers was used as a measure of the change in synaptic density.

### Statistical analysis

All data are expressed as the mean ± S.D. For MWM learning test, we used univariate repeated measures ANOVA to analyze the data. Otherwise, without specific indication, comparisons of the mean values between the two groups were performed using Student's *t*-test. Statistically significant differences were identified when p<0.05.

## Results

### Age-dependent intraneuronal accumulation of Aβ oligomers without extracellular amyloid plaques in PS1_V97L_-Tg mice

**Figure 1:**
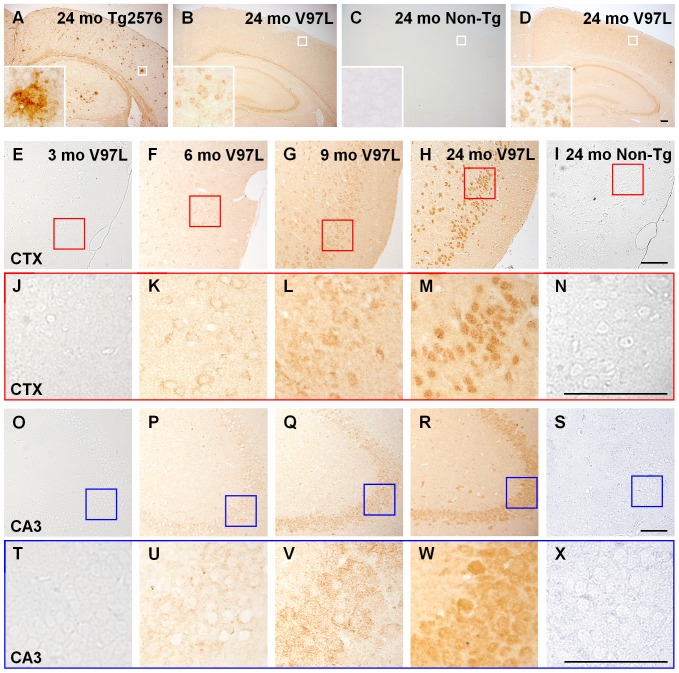
Age-dependent accumulation of Aβ oligomers in the neurons of PS1_V97L_-Tg mice. (**A, B, C**) Brain sections probed by antibody 4G8 reflect PS1_V97L_-Tg mice exhibiting intracellular Aβ protein, without extracellular amyloid plaque. (**D**) Brain sections probed by antibody A11 reflecting PS1_V97L_-Tg mice exhibiting intracellular Aβ oligomers. (**E–X**) Display showing the age-dependent accumulation of Aβ oligomers stained with A11. PS1_V97L_-Tg is presented as V97L for short. CTX, cerebral cortex; CA3, hippocampal CA3 region. Note that (**J–N**) and (**T–X**) are higher magnifications of (**E–I**) and (**O–S**), respectively. Scale bar represents 100 µm.

Initially, we examined the brain amyloid plaques in PS1_V97L_-Tg mice by immunohistochemistry using an Aβ specific antibody (4G8). Although Tg2576 mice, which we used as a positive control, displayed abundant extracellular amyloid plaques, the PS1_V97L_-Tg mice exhibited no amyloid plaques, but showed intracellular positive stain in the cerebral cortex or the hippocampus, at 24 months of age. The Non-Tg littermates exhibited no Aβ staining in any region (**Figure 1A, B, C**). Further detected with Aβ oligomer specific antibody (A11), the PS1_V97L_-Tg mice exhibited abundant intraneuronal staining in the cerebral cortex and hippocampus (**Figure 1D**).

Temporal and regional profiles showed that in the brains of PS1_V97L_-Tg mice, intraneuronal Aβ oligomers were undetectable at 3 months (**Figure 1E, J, O, T**); appeared at 6 months in specific layers of the cerebral cortex, particularly the entorhinal region (**Figure 1F, K**); progressed to the hippocampus by 9 months (**Figure 1G, L, Q, V**); accumulated in an age-dependent fashion; and finally emerged as a heavily stained mass at 24 months (**Figure 1H, M, R, W**). No Aβ oligomer staining was observed in Non-Tg littermates (**Figure 1I, N, S, X**) even at 24 months.

We observed intraneuronal Aβ oligomer accumulation to various extents in other brain regions, including the thalamus, cerebellum and brain stem. Aβ oligomer accumulation in mice as old as 30 months did not significantly exceed that in 24-month-old mice. Due to space limitations, here we report the results of the cortex and hippocampus as representative. Our time-series represents the most relevant time points: a time point 3 months before abnormalities appeared, a time point when the abnormalities first appeared, subsequent time points paralleling behavioral changes and a comparatively old age (i.e., 24 months). The time-points thus establish pre-pathological characteristics of each group, the emergence of abnormalities, and the outcomes of the late stage of disease. We collected complete progression trends occurring in the PS1_V97L_-Tg mouse model. The same procedures were followed with regards to the results of other pathologies.

**Figure 2:**
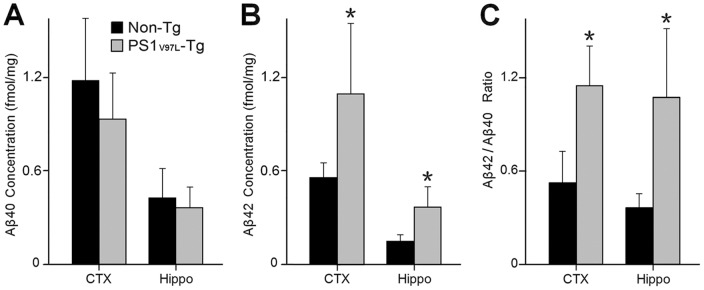
Increases in the levels of Aβ42 and the ratio of Aβ42/Aβ40 in PS1_V97L_-Tg mice at 9 months of age. (**A**) Aβ40 expression level. (**B**) Aβ42 expression levels. (**C**) The ratio of Aβ42/Aβ40. ELISA measurements are from the cortex and the hippocampus of 9-month-old mice. * denotes a significant difference at p<0.05 (n = 6/group).

To determine the content and constitution of the Aβ species, brain homogenates of the cortex and the hippocampus from PS1_V97L_-Tg mice were subjected to ELISA assays to measure the levels of Aβ40 and Aβ42. Compared with the Non-Tg littermates, the Aβ40 content in PS1_V97L_-Tg mice was not significantly altered in the cortex or the hippocampus (p>0.05) (**Figure 2A**), but Aβ42 content in PS1_V97L_-Tg mice increased in both areas at 9 months (p<0.05) (**Figure 2B**). Thus, there was a significant increase in the Aβ42/Aβ40 ratio compared with the Non-Tg control group (p<0.05) (**Figure 2C**).

### Memory and synaptic dysfunction in PS1_V97L_-Tg mice

**Figure 3:**
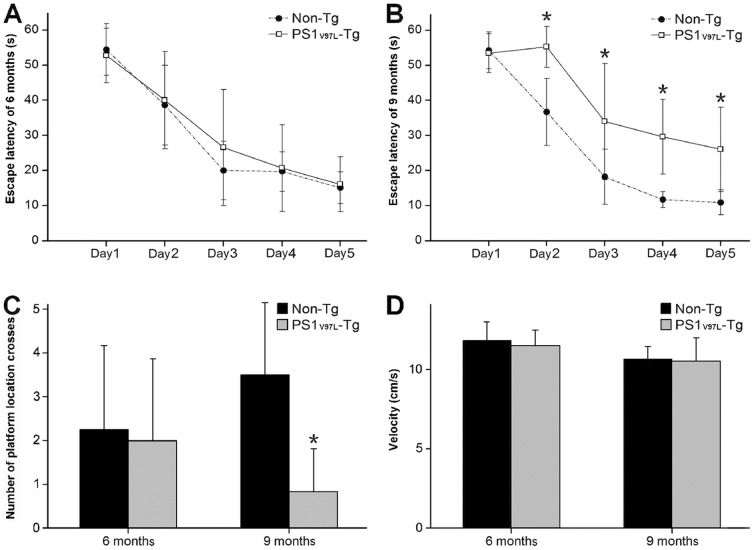
Memory dysfunction in 9-month-old PS1_V97L_-Tg mice. (**A**) MWM escape latency during training at 6 months or (**B**) at 9 months. (**C**) Numbers of platform location crosses in the probe trials at 6 and 9 months. (**D**) Swimming speed on the first training day at 6 and 9 months. * indicates a significant difference at p<0.05 (n = 6/group).

We assessed spatial learning and memory retention in 6- and 9-month-old PS1_V97L_-Tg and Non-Tg mice using the MWM. In the spatial learning test at 6 months, there was no significant difference in escape latency between the PS1_V97L_-Tg and Non-Tg groups (p>0.05) (**Figure 3A**). The escape latency in the 9-month-old PS1_V97L_-Tg mice was significantly longer than in the age-matched Non-Tg littermates after the second training day (p<0.05) (**Figure 3B**). In the probe trials after 5 training days, we found that the PS1_V97L_-Tg mice crossed the platform location less often than the Non-Tg group at 9 months (p<0.05); at 6 months, the two groups were not significantly different (p>0.05) (**Figure 3C**). In addition, we did not find any difference in swimming speed on the first training day between the two groups at either 6 or 9 months (p>0.05), which excludes any potential influence of motor disabilities on escape latency (**Figure 3D**).

**Figure 4:**
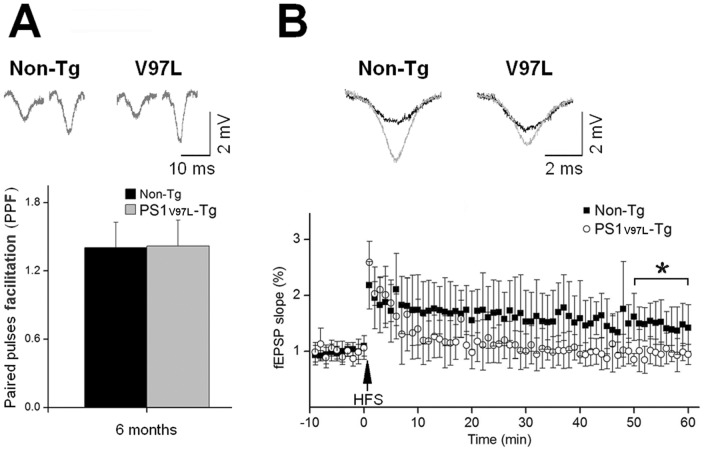
Impairment of synaptic plasticity in hippocampal slices from 6-month-old PS1_V97L_-Tg mice. (**A**) Comparison of PPF in 6-month-old mice. The upper channel shows the typical fEPSP traces derived from paired stimuli with an interpulse interval of 20 ms. The bottom channel shows the PPF in PS1_V97L_-Tg and Non-Tg mice. (**B**) Comparison of LTP in 6-month-old mice. The upper channel shows the typical fEPSP traces before (black) and 1 h after (grey) HFS delivery. The bottom channel shows the slopes of the fEPSPs during the 70 min study. The slopes recorded in the last 10 min were analyzed; * indicates a statistically significant difference at p<0.05 (n = 10/group). Note that artifacts are not shown.

Synaptic plasticity was examined in hippocampal slices from the PS1_V97L_-Tg and Non-Tg mice. Paired-pulse facilitation (PPF) of field excitatory postsynaptic potentials (fEPSPs) at the CA3-CA1 synapses was elicited in Schaffer collateral (SC) fibers after paired stimuli with a 20 ms interpulse interval. At 6 months of age, prior to the memory deficit detected by MWM, slices from the Non-Tg and PS1_V97L_-Tg mice showed significant facilitation of the 2nd fEPSP evoked by the paired stimuli (**Figure 4A**). The average facilitation was 143±16.6% (Non-Tg) and 146±15.6% (PS1_V97L_-Tg), indicating that short-term plasticity was not influenced by the mutation. However, an attenuation of long-term potentiation (LTP) was observed in the SC-CA1 pathway in the 6-month-old PS1_V97L_-Tg mice; the fEPSPs showed a more rapid rundown and retained a lower plateau after high frequency stimulation (HFS) compared with Non-Tg mice (**Figure 4B**). These findings suggest that long-term plasticity in hippocampus of PS1_V97L_-Tg mice has already been functionally impaired prior to the observed memory decline.

**Figure 5:**
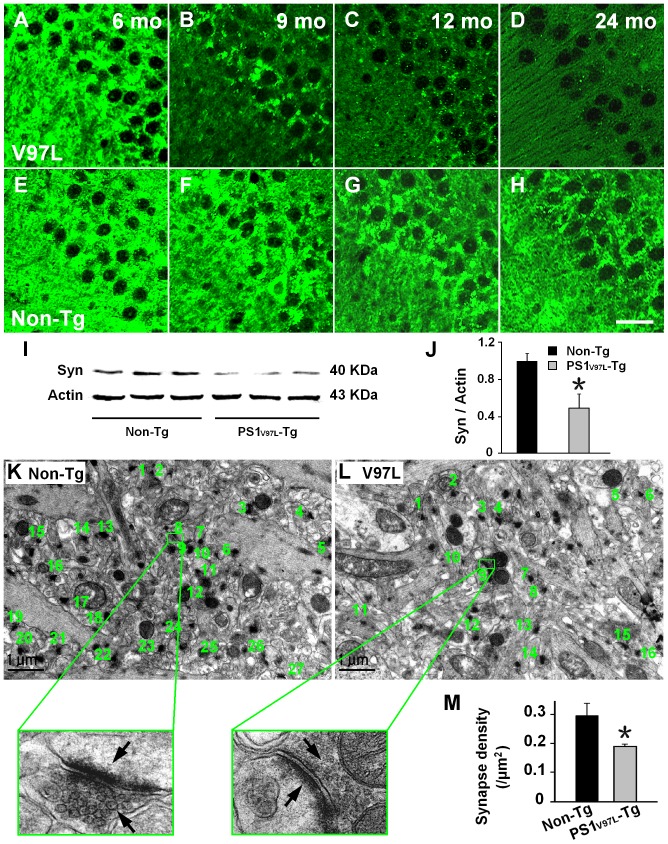
Age-dependent reduction in synapse number in 9-month-old PS1_V97L_-Tg Mice. (**A–H**) Brain sections from the hippocampal CA3 region of PS1_V97L_-Tg mice at different ages probed with an antibody to the presynaptic marker synaptophysin. Scale bar represents 20 µm. (**I, J**) Expression levels of synaptophysin in the hippocampus of PS1_V97L_-Tg mice were significantly reduced compared with Non-Tg littermates tested by western blotting at 9 months (p<0.05, n = 3/group) and is indicated by an asterisk. (**K, L**) Representative electron microscope photographs taken from the neuropil region of the CA3 in 12-month-old Non-Tg and PS1_V97L_-Tg mice. Synapses were identified by the presence of synaptic vesicles and postsynaptic densities, which are typically shown at high magnification and are indicated by arrows. (M) Comparison of synapse density between the PS1_V97L_-Tg mice and Non-Tg littermates showed a significant difference (p<0.05, n = 6/group) and is indicated by an asterisk.

To examine whether synaptic loss occurred in the PS1_V97L_-Tg mice, brain sections from mice at various ages were stained with presynaptic marker synaptophysin. There was no apparent decrease of synaptophysin at 6months old in PS1_V97L_-Tg mice (**Figure 5A, E**). However, compared with Non-Tg littermates, PS1_V97L_-Tg mice exhibited a significant decrease in synaptophysin in the hippocampus, particularly in the CA3 region, in an age-dependent fashion from 9 months on (**Figure 5A-H**). Western blotting confirmed this decrease in synaptophysin expression at 9 month (p<0.05) (**Figure 5I, J**). A significant decrease in synaptic density in PS1_V97L_-Tg mice was detected by electron microscopy by 12 months compared with Non-Tg littermates (p<0.05) (**Figure 5K-M**).

We did not use positive cell count or densitometry analysis as quantitative measures of each intended target (e.g., synaptophysin) in our experiment, as these measures would additionally require stereological analysis. Considering the feasibility of the experiment and that no distinctive difference in distribution of our intended targets was observed in immunochemistry tests, we chose western blotting to depict the semi-quantitative difference between the two groups. We chose the 9-month-old time point for western blotting because the first detectable significant difference between the two groups occurred at this time. We also tested the 6-month-old mice (no difference between the two groups in the present testing system) and 12-month-old mice (more obvious differences; however, not the first time point of detectable difference) in the preliminary experiment. For the above reasons, and due to space limitations, we report only the time points relevant for distinguishing the groups and the temporal progression of their disease.

### Tau hyperphosphorylation in PS1_V97L_-Tg mice

**Figure 6:**
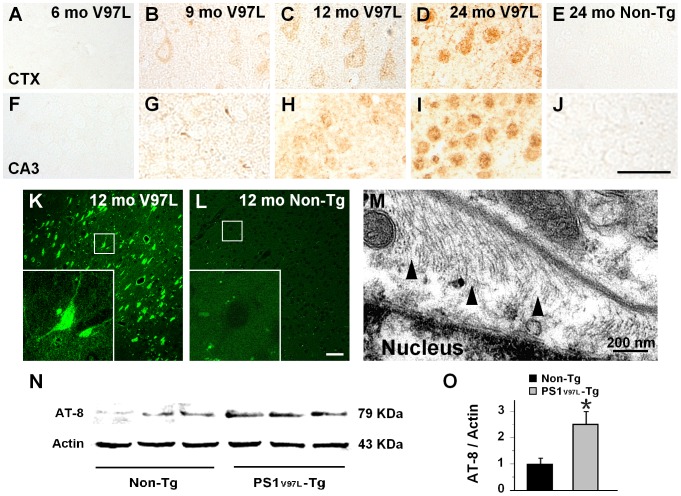
Tau hyperphosphorylation and tangle formation in PS1_V97L_-Tg mice. (**A–J**) Detailed graphs of the cortex and hippocampus that show the age-dependent intracellular accumulation of AT-8 staining. (K, L) High-magnification of cortex from 12-month-old PS1_V97L_-Tg mice stained with Thioflavin-S, which indicates that intracellular hyperphosphorylated tau forms NFTs compared with its Non-Tg littermate. (M) A representative electron microscope photograph revealing the presence of tau filaments that formed an herringbone pattern (black arrows) in 12-month-old PS1_V97L_-Tg mice. (**N, O**) Western blotting analysis of AT-8 expression in the cerebral cortex of 12-month-old PS1_V97L_-Tg mice and Non-Tg littermates. An asterisk indicates significant difference between the two groups (p<0.05, n = 3/group). CTX, cerebral cortex. Scale bar represents 50 µm for (**A–L**).

We investigated abnormal tau phosphorylation in the PS1_V97L_-Tg mice. Based on our previous study that investigated several sites of tau hyperphosphorylation [15], we chose a representative site Ser202 to test the progression of tau hyperphosphorylation. Brain sections from mice of various ages were stained using AT-8, a specific antibody that stains Ser202 and pathologically phosphorylated tau [22], [23]. There was no AT-8 positive staining in the 6 months old PS1_V97L_-Tg mice brain (**Figure 6A, F**). It began to exhibit tau hyperphosphorylation in cortical neurons at 9 months (**Figure 6B**). From 12 months on, tau hyperphosphorylation was more pronounced (**Figure 6C, H**). Tau hyperphosphorylation increased with aging (**Figure 6D, I**). In contrast, the Non-Tg littermates exhibited no hyperphosphorylated tau staining in any examined regions even at 24 months (**Figure 6E, J**).

Furthermore, to confirm the tau hyperphosphorylation, we probed the brain sections with Thioflavin-S staining. This staining showed intracellular NFT formation in PS1_V97L_-Tg mice (**Figure 6K, L**). Electron microscopic examination revealed intracellular tau filaments (**Figure 6M**) close to neuronal nuclei, indicating hyperphosphorylated tau in NFTs. Western blotting analysis showed an apparent increase in the levels of AT-8 immunoreactivity in the cortex of PS1_V97L_-Tg mice compared with the Non-Tg control at 12 months of age (p<0.05) (**Figure 6N, O**).

### Glial activation in PS1_V97L_-Tg mice

**Figure 7:**
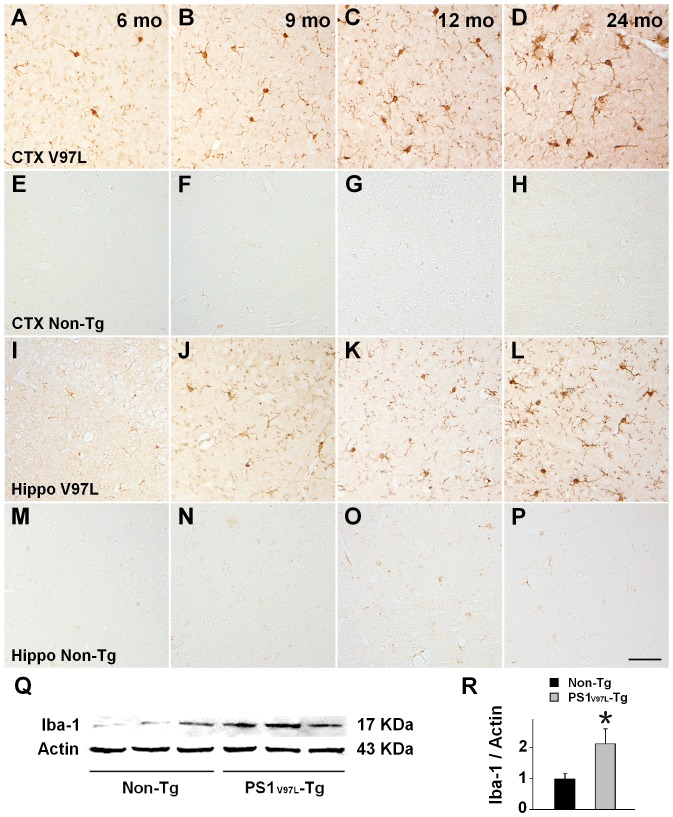
Microglial activation in PS1_V97L_-Tg mice. (**A–H**) Brain sections taken from the cerebral cortex and probed by an antibody to Iba-1, indicating activated microglial cells. (**I–P**) Brain sections taken from the hippocampal CA3 region probed by an antibody to Iba-1, indicating activated microglial cells. (**Q, R**) Iba-1 expression in the cerebral cortex of PS1_V97L_-Tg mice and Non-Tg littermates at 9 months, tested by western blotting. An asterisk indicates significant difference between the two groups (p<0.05, n = 3/group). CTX, cerebral cortex; Hippo, hippocampus. Scale bar represents 50 µm.

Finally, we examined glial activation. Brain sections from mice of various ages, including the cortex and the hippocampus, were stained with an antibody to Iba-1, which is a marker of activated microglia. Non-Tg littermates exhibited a very low active level of microglia even at 24 months (**Figure 7** **E–H, M–P**). In contrast, PS1_V97L_-Tg mice began to display activated microglia at 6 months, and by 9 months, the activated microglia were obvious in the cortex and the hippocampus (**Figure 7A–D, I–L**). Western blotting analysis confirmed that the levels of microglial activation in the PS1_V97L_-Tg mice significantly exceeded the Non-Tg mice at 9 months (p<0.05) (**Figure 7Q, R**). Similar results were obtained for astrocytes stained with a specific antibody, GFAP (**Figure S1**).

Normally, glial activation is the reactive response when the brain experiences any damage, for example, ischemia, trauma, deprivation of oxygen or glucose, exposure to toxic bacterial or viral species and toxic metabolism. Therefore, we did not attempt to identify the first time point to show abnormality, as shown in the series. We took glial activation to be an accompanying event in response to the toxic Aβ oligomer. Therefore, we chose 6 months as the starting point to show the disease progression.

## Discussion

In this study, following intraneuronal accumulation of Aβ oligomers which were probably caused by PS1 V97L mutation, we detected synaptic alterations, memory dysfunction, abnormal tau phosphorylation and glial activation in the absence of amyloid plaques in PS1_V97L_-Tg mice. These findings suggest that Aβ oligomers are likely to play an initiating role in the onset and development of AD; amyloid plaque formation may not be an absolute requirement.

The amyloid cascade hypothesis, the best defined and most studied of the various theories for AD, emerged in the late 1980s [24], [25]. It was proposed that the deposition of Aβ, particularly its fibrillar form, which is the main component of the plaques, was the causative agent for AD and that other Alzheimer pathology follows as a direct result of this deposition [26]. Cell biology provides further support for the amyloid cascade hypothesis by linking Aβ assemblies to neurotoxicity [27]–[30]. The common forms of Aβ include Aβ monomers, Aβ oligomers and Aβ fibrils. Monomeric Aβ itself is innocuous to cultured neurons, but it becomes neurotoxic upon self-association [30]. Aβ fibrils can cause significant synapse reduction in viable neurons in primary hippocampal culture [28], [29]. More recently, the dominant hypothesis for the pathogenesis of AD has undergone revision, primarily in relation to the pathogenic forms of Aβ [31], [32]. The evolution is based on the “oligomer hypothesis,” which suggests that the most toxic species is not the fibrillar aggregate but the oligomeric Aβ [33]. Our current PS1_V97L_-Tg mouse model favors this hypothesis: AD progression in the absence of pathological plaques and the neuronal accumulation of oligomers support the view that oligomeric Aβ rather than amyloid plaques are the critical toxic species.

In the brains of PS1_V97L_-Tg mice, we found Aβ oligomers within neurons at 6 months; these became more evident as age increased. This is the first striking pathologic event observed in this model. It is possible that the presence of Aβ oligomers might be the result of the mutation of the PS1 gene, per se, and the causal relationship between PS1 gene mutations and the presence of Aβ oligomers cannot be completely clarified by the present experiment; this issue will need to be explored in further studies. It now appears that Aβ oligomers are the most pathogenically relevant Aβ-derived toxins [34], [35]. Other groups also found the existence of intracellular oligomers in different transgenic animal models [36], [37]. We wondered why there was no amyloid plaque in this model. In transgenic mice based on the human FAD PS mutation, Aβ-dependent effects usually result from overproduction of mouse-sourced Aβ, which has different aggregation properties than human Aβ [38], specifically its tendency not to form fibrillar aggregates. These differences might underlie the absence of plaque in PS1_V97L_-Tg mice. Next, in an attempt to provide explanations for the Aβ oligomer accumulation in a large population of neurons, we analyzed the content and constitution of the Aβ species, chiefly Aβ40 and Aβ42, in the PS1_V97L_-Tg mice. We found that by 9 months, there were significant increases in the Aβ42 content and the Aβ42/Aβ40 ratio, which have been suggested to be conditions that favor oligomerization [39], [40]. Our findings may suggest that changes in Aβ species in the PS1_V97L_-Tg mice could increase the accumulation of Aβ oligomers in neurons. However, we cannot exclude the involvement of other mechanisms, such as reduced degradation, and this needs to be clarified by further investigation.

Overall, we have interpreted the 4G8 and A11 signal as evidence for intracellular oligomeric Aβ. In fact, A11 is a conformation-specific antibody that detects an epitope shared by aggregates of many different proteins, including, α-synuclein, insulin, and prion protein. But A11 can only bind with Aβ oligomers instead of monomers and fibrils [41]. Further biochemical analysis is required to prove the A11 identity in our experiment. However, A11 is an antibody known to require specific conditions (total or nearly total absence of detergent/SDS) to detect its intended target, which makes western blotting unreliable [34]. On the other hand, 4G8 stain showed intracellular positive stain, which means PS1_V97L_-Tg mice has intracellular Aβ protein. Further detecting by A11 antibody reassured that the intracellular Aβ protein were Aβ oligomers, rather than monomers or fibrils.

Following Aβ oligomer accumulation in the neurons of PS1_V97L_-Tg mice, synaptic loss and memory dysfunction emerged. Therefore, we postulate that synapse loss and memory dysfunction might be caused by Aβ oligomers, without the necessity of amyloid plaque formation. Other studies that have reached similar conclusions using different experimental approaches support our view. Lacor et al. found that synthetic Aβ-derived oligomers (ADDLs) might cause a significant decrease in spine density in highly differentiated cultures of hippocampal neurons [42]. By comparing several APP Tg-mice, Mucke and colleagues stated that decreases in synaptophysin, which indicated synaptic loss, were not dependent on plaque formation [43]. Moreover, memory formation is impaired by intracerebral injections of oligomers, whether made *in vitro* or *in vivo* [44]. Additionally, different groups have independently found that memory loss was reversed in APP-Tg mice injected with Aβ antagonists. This reversal of memory loss, which is achieved by neutralizing Aβ assemblies in the brain, did not accompany the removal of amyloid plaques [45], [46]. Furthermore, some neuropathologists have argued that the correlation between cognitive function in AD patients and their plaque load at postmortem examination is poor [47], [48], indicating that there must be something else other than the amyloid plaques that can explaining the mechanism of onset and the development of AD. Although amyloid plaques were not the agent, the accumulation of Aβ oligomers, which was the earliest event detected in our model, might be essential for synaptic loss and the subsequent memory dysfunction.

Interestingly, prior to memory dysfunction, we observed the early inhibition of LTP measured in brain slices from PS1_V97L_-Tg mice at 6 months; short-term plasticity was unchanged at this age, which suggested that baseline excitability was not impaired. These findings indicate the synaptic impairment of signaling rather than structural degeneration. This phenomenon occurred in parallel with the intracellular accumulation of Aβ oligomers when there was no detectable intracellular tau hyperphosphorylation, indicating that Aβ oligomers might be synaptotoxic and probably account for the initiation of memory dysfunction in this mouse model rather than tau which needs to be clarified by further study. These findings lend *in situ* support to the “oligomer hypothesis”: memory loss was caused by Aβ oligomer-induced disruption of synaptic plasticity. The oligomer hypothesis was proposed based on experimental evidence that synthetic Aβ oligomers induce impairment of synaptic plasticity [44], [49], [50], memory dysfunction [50]–[53] and synaptic loss [54] when applied exogenously to dissociated neurons, cultured brain slices, or the rat cerebral ventricle. Here, we provide another useful tool in exploring the Aβ oligomer hypothesis *in vivo*, in addition to the APP single mutation transgenic mouse model APP_E693_-Tg, which also exhibited intraneuronal Aβ oligomers without extracellular plaques but failed to form NFTs [37]. Oligomers might cause synaptic dysfunction and impaired memory formation, which arguably can account for the primary aspect of dementia in early AD. Oligomers can also be linked to other major facets of AD neuropathology.

The most popular recent area of research in the field of AD pathogenesis is the link between Aβ oligomers and tau pathology. We investigated the age-dependent sequential appearance of pathology in an attempt to determine whether there is a potential causal relationship between Aβ oligomers and tau hyperphosphorylation in PS1_V97L_-Tg mice. We examined tau pathology by immunohistochemistry, Thioflavin-S staining and ultrastructure analysis using electron microscopy. PS1_V97L_-Tg mice displayed abnormal tau hyperphosphorylation in the cerebral cortex starting at 9 months of age, 3 months after the intraneuronal accumulation of Aβ oligomers was observed. Thus, our findings suggest that Aβ oligomers may induce tau pathology *in vivo*. These findings are consistent with several other studies that demonstrate that the neurotoxicity observed with the different Aβ oligomer preparations is associated with enhanced tau phosphorylation and can be attenuated when the tau gene is silenced or knocked out [55]–[57]. Another group demonstrated an interaction between tau and Aβ oligomers, where they accelerate each other's aggregation [58]. Thus, our findings suggest that there is high probability that Aβ oligomers initially induce tau hyperphosphorylation and then pathologic tau mediates the neurotoxicity of Aβ oligomers, a suggestion consistent with earlier observations in APP23 mice in which the pathologic function of tau in dendrites may disturb N-methyl-D-aspartate (NMDA) receptors and confer Aβ toxicity [59].

Activation of glial cells is an important characteristic of inflammation that is usually involved in the progression of AD [60]. Clustering of activated microglia and astrocytes around amyloid plaques was detected in neurodegeneration [61]. Under immunohistochemical examination, the PS1_V97L_-Tg mice exhibited a few activated microglia and astrocytes (**Figure S1**) at 6 months accompanying the accumulation of Aβ oligomers; activated microglia were more evident at 9 months old in the cerebral cortex and hippocampus, an observation that was confirmed by Western blot analysis. Although earlier studies have focused on glial cell activation after amyloid plaque formation, in the PS1_V97L_-Tg mice, glial cells appear to be recruited toward neurons with intracellular Aβ oligomers without the involvement of amyloid plaques. A possible explanation is that glial cells have been activated and recruited by cytokines or chemokines released from neighboring glial cells that were in contact with the aberrant neurons containing Aβ oligomers. It is also possible that the glial reaction observed in the plaque-free PS1_V97L_-Tg mice is triggered by factors that have diffused from damaged or dying Aβ-burdened neurons. In fact, it is well known that unhealthy neurons can release a variety of signaling molecules that microglia respond to [62], [63]. A similar recruitment of microglial cells towards neurons has been demonstrated recently using 2-photon *in vivo* imaging technology in a mouse model of AD [64].

There was no significant trend of neuronal loss in the CA3 region or a specific layer of the cerebral cortex even in 24-month-old PS1_V97L_-Tg mice. This might result from the protective activity of glial cells; however, the limited relevant evidence we have thus far is insufficient to claim this with any certainty. Moreover, we occasionally found obvious neuronal loss with ventricular enlargement compared with these animals' Non-Tg littermates (**Figure S2**) (in 36 mice that underwent morphological tests, we found only 2 with obvious brain atrophy: one was 14 months old and the other was 10 months old). We hope to address this specific issue in a further study.

We are now treating PS1_WT_-Tg mice, as reported in Wang et al.'s study [15], as another (less successful) PS1 transgenic mouse model, which overexpresses the human wild-type PS1 gene. We also included PS1_WT_-Tg mice in the preliminary experimental protocol. We carried out behavioral tests using the MWM, immunohistochemistry tests using 4G8, A11, and AT-8, and an ELISA for the content of Aβ40 and Aβ42 in the brain. The animals had no learning deficiency at 9 months of age (**Figure S3**) and intracellular expression of A11 was first detected in the cortex at 9 months, which increased more slowly with age compared with PS1_V97L_-Tg mice (**Figure S4**). However, there was no significant difference of Aβ40 and Aβ42 expression in the brain as compared with Non-Tg littermates by the age of 9 months (p>0.05) (**Figure S5**), and no AT-8 expression in the brain even at 24 months (**Figure S6**). On the basis of our results, we drew the conclusion that the V97L mutation in the PS1 gene brings about more robust pathological features than simply the overexpression of the PS1 gene *per se*. Therefore, we chose to study the PS1_V97L_-Tg mice as our novel model.

In summary, because of the PS1 gene V97L mutation, PS1_V97L_-Tg mice developed a series of AD-typical pathological characteristics, which were initiated by an accumulation of intraneuronal Aβ oligomers, and in the absence of extracellular plaques. The model used excludes interference effects from extracellular plaques on the effects of Aβ oligomers, which is a problem encountered in many studies of the mechanisms of action of Aβ oligomers in AD. However, the precise molecular identity of Aβ oligomers that account for the toxicity in PS1_V97L_-Tg mice is unknown, and thus, further research is needed. Nevertheless, in addition to directly affecting memory-related processes, the various impacts of Aβ oligomers on neurons might have the potential to account for major facets of AD neuropathology (for example, tau hyperphosphorylation, glial activation and synapse loss) supporting the concept that Aβ oligomers provide a unifying mechanism for initiation of AD pathogenesis. The establishment of PS1_V97L_-Tg mice provides a useful *in vivo* tool for explore the Aβ oligomer hypothesis, which might be a potential target for AD drug development.

## Supporting Information

Figure S1 **Astrocyte activation in PS1_V97L_-Tg mice.** (A–H) Brain sections taken from cerebral cortex probed by antibody GFAP indicating activated astrocytes. (I–P) Brain sections taken from hippocampal CA3 region probed by antibody GFAP indicating activated astrocytes. (Q, R) GFAP expression in PS1_V97L_-Tg mice and Non-Tg littermates cortex in the 9th month tested by western blotting. An asterisk indicates significant difference between the two groups (p<0.05, n = 3/group). CTX, cerebral cortex; Hippo, hippocampus. Scale bar represents 50 µm.
(TIF)

Figure S2 **Occasionally found neuronal loss with ventricular enlargement in PS1_V97L_-Tg mice.** (A, D) Brain sections probed using the NeuN antibody are from 14-month-old PS1_V97L_-Tg mice exhibiting obvious brain atrophy with ventricular enlargement compared with their Non-Tg littermates. (B, E) and (C, F) are higher magnifications of the red and blue windows, separately. CTX, cerebral cortex; Hippo, hippocampus.
(TIF)

Figure S3 **PS1_WT_-Tg mice showed no impaired spatial learning and memory at 9 months of age.** Escape latency of mice in different groups at training stage of five days. * indicates a significant difference at p<0.05, PS1_V97L_-Tg vs. Non-Tg littermates; as to PS1_WT_-Tg vs. Non-Tg littermates, no significant difference was detected (p>0.05) (n = 6/group).
(TIF)

Figure S4 **Accumulation of Aβ oligomers in the neurons of PS1 _WT_-Tg mice.** This figure shows the accumulation of Aβ oligomers stained with A11. PS1_WT_-Tg is presented as PS1 for short. It is detected that PS1_WT_-Tg mice, which beard human PSEN-1 wild type insertion, developed accumulation of Aβ oligomers in neurons, but progressed more slowly than PS1_V97L_-Tg mice (Figure 1 in manuscript). It suggested that V97L mutation could obviously promote generation and accumulation of Aβ oligomers in an early stage. CTX, cerebral cortex; CA3, hippocampal CA3 region. Note that (F–J) and (P–T) are higher magnifications of (A–E) and (K–O), respectively. Scale bar represents 100 µm.
(TIF)

Figure S5 **No change in the levels of Aβ40 and Aβ42 in PS1_WT_-Tg mice at 9 months.** (A) Aβ40 expression level. (B) Aβ42 expression levels. (C) The ratio of Aβ42/Aβ40. ELISA measurements are from the cortex and the hippocampus of 9-month-old mice. * denotes a significant difference at p<0.05 vs Non-Tg littermates (n = 6/group).
(TIF)

Figure S6 **No positive abnormal tau hyperphosphorylation stain in PS1_WT_-Tg mice even at 24 months.** (A–F) Detailed graphs of the cortex and hippocampus that show no intracellular accumulation of AT-8 staining in PS1_WT_-Tg mice, compared with positive control PS1_V97L_-Tg mice. Scale bar represents 50 µm.
(TIF)
