# Supplementary figures and images for: A Lifespan Observation of a Novel Mouse Model: In Vivo Evidence Supports Aβ Oligomer Hypothesis
Source: PLoS One. 2014 Jan 21;9(1):e85885. doi: 10.1371/journal.pone.0085885 (PMC3897547; doi:10.1371/journal.pone.0085885)

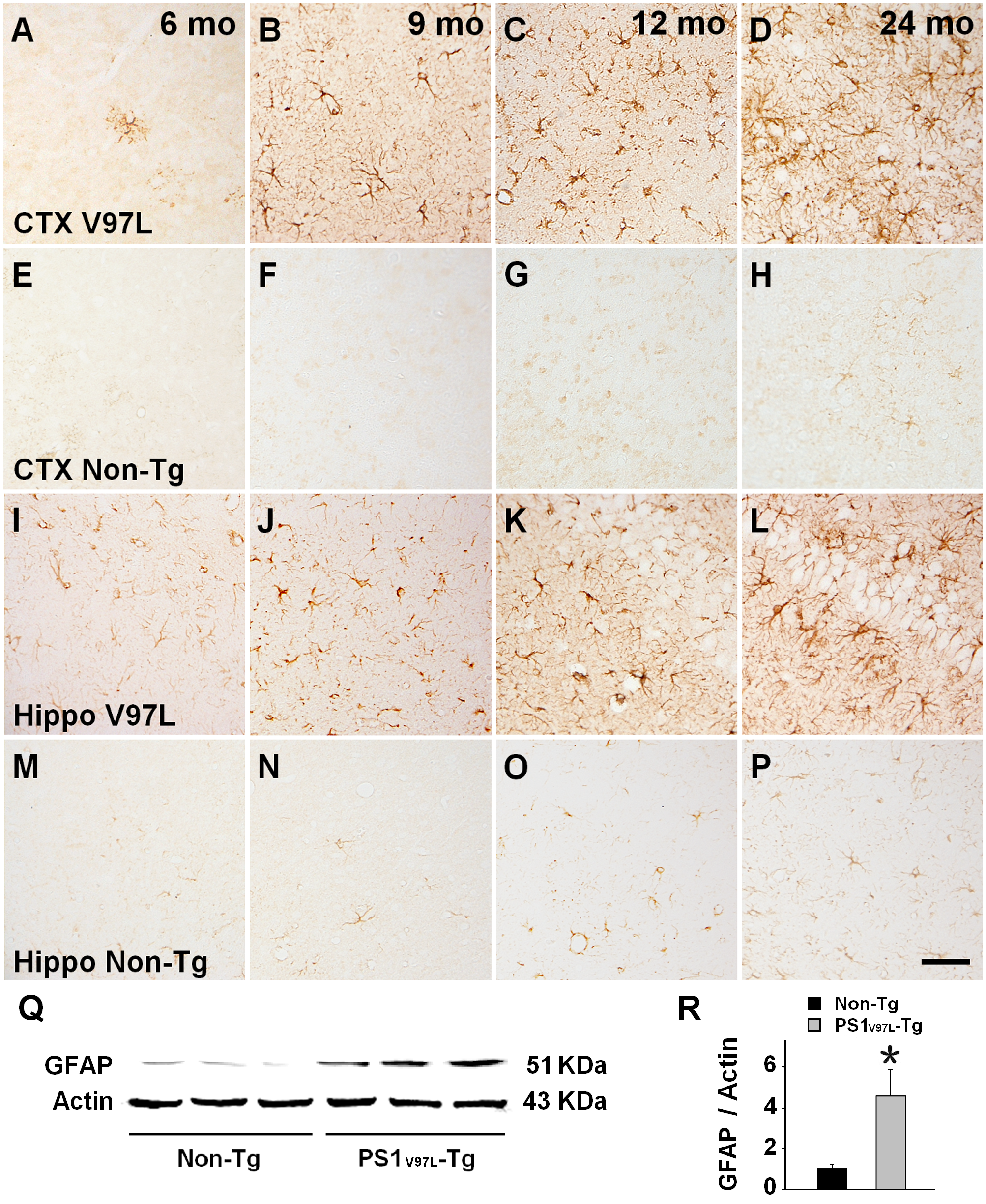

Supplement: Figure S1 — Astrocyte activation in PS1V97L-Tg mice. (A–H) Brain sections taken from cerebral cortex probed by antibody GFAP indicating activated astrocytes. (I–P) Brain sections taken from hippocampal CA3 region probed by antibody GFAP indicating activated astrocytes. (Q, R) GFAP expression in PS1V97L-Tg mice and Non-Tg littermates cortex in the 9th month tested by western blotting. An asterisk indicates significant difference between the two groups (p<0.05, n = 3/group). CTX, cerebral cortex; Hippo, hippocampus. Scale bar represents 50 µm. (TIF) [file pone.0085885.s001.tif]

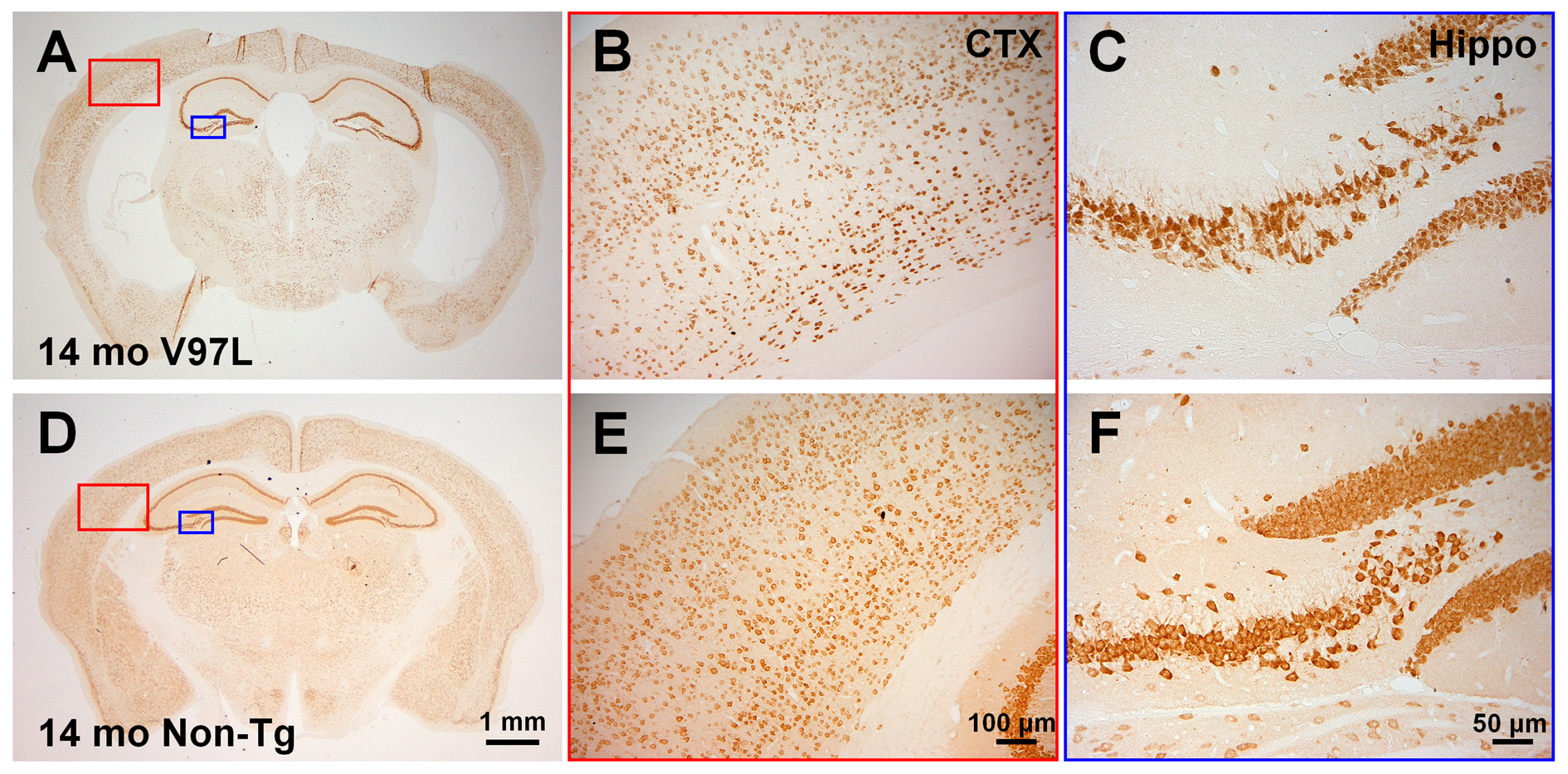

Supplement: Figure S2 — Occasionally found neuronal loss with ventricular enlargement in PS1V97L-Tg mice. (A, D) Brain sections probed using the NeuN antibody are from 14-month-old PS1V97L-Tg mice exhibiting obvious brain atrophy with ventricular enlargement compared with their Non-Tg littermates. (B, E) and (C, F) are higher magnifications of the red and blue windows, separately. CTX, cerebral cortex; Hippo, hippocampus. (TIF) [file pone.0085885.s002.tif]

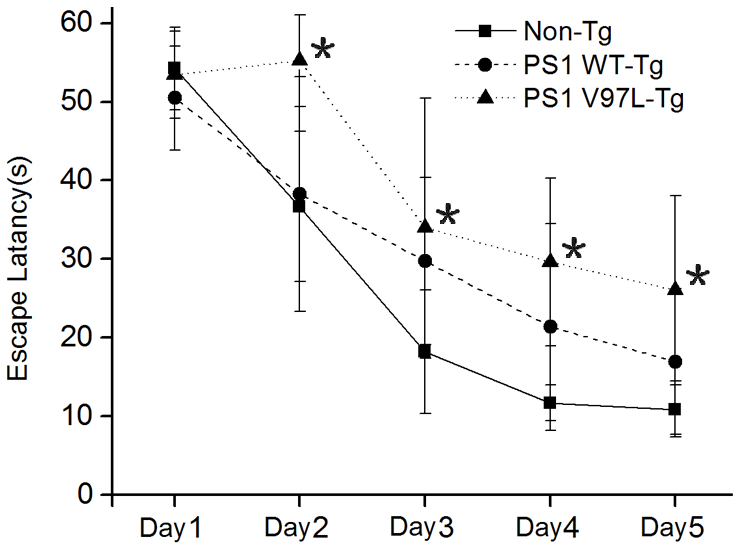

Supplement: Figure S3 — PS1WT-Tg mice showed no impaired spatial learning and memory at 9 months of age. Escape latency of mice in different groups at training stage of five days. * indicates a significant difference at p<0.05, PS1V97L-Tg vs. Non-Tg littermates; as to PS1WT-Tg vs. Non-Tg littermates, no significant difference was detected (p>0.05) (n = 6/group). (TIF) [file pone.0085885.s003.tif]

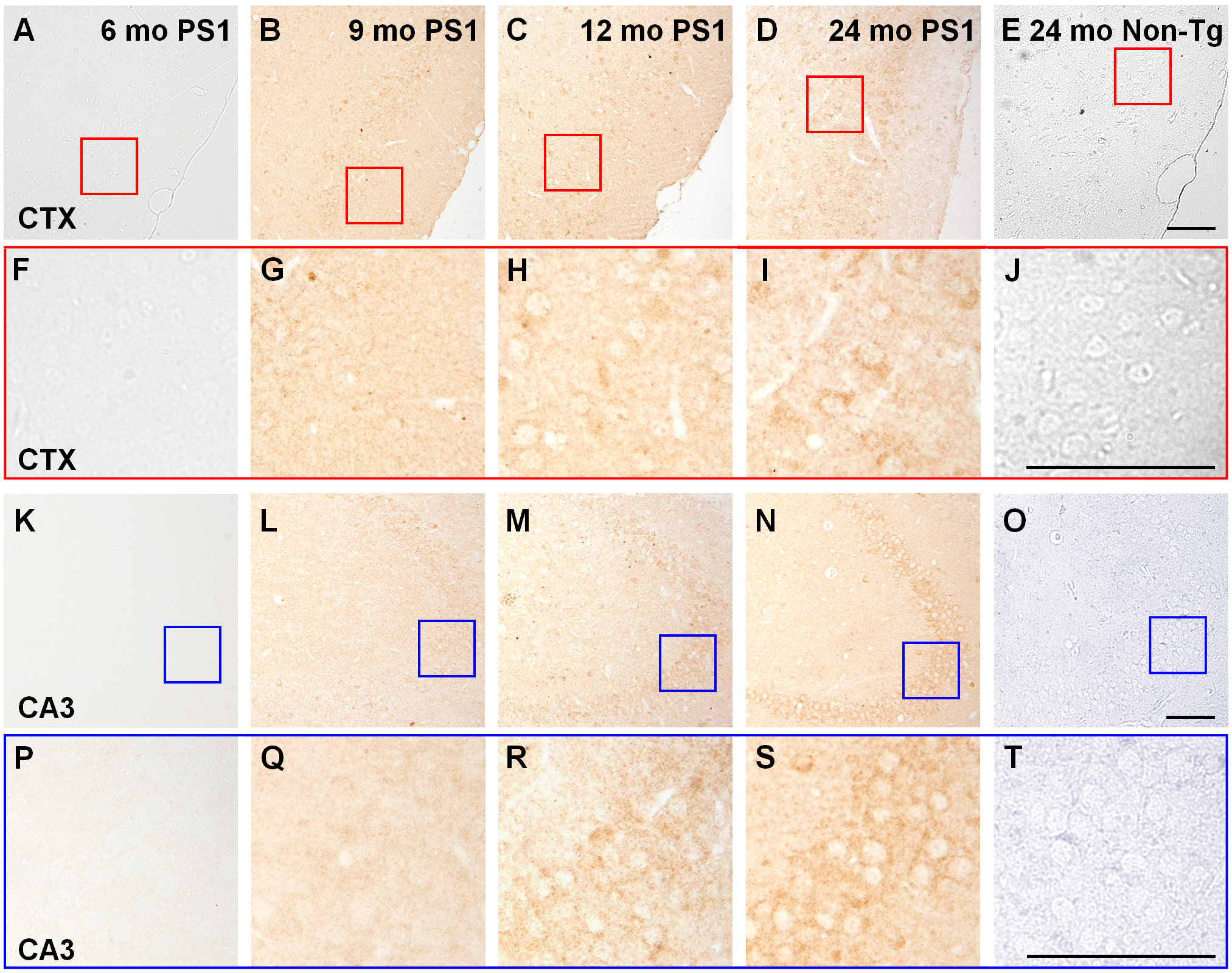

Supplement: Figure S4 — Accumulation of Aβ oligomers in the neurons of PS1 WT-Tg mice. This figure shows the accumulation of Aβ oligomers stained with A11. PS1WT-Tg is presented as PS1 for short. It is detected that PS1WT-Tg mice, which beard human PSEN-1 wild type insertion, developed accumulation of Aβ oligomers in neurons, but progressed more slowly than PS1V97L-Tg mice (Figure 1 in manuscript). It suggested that V97L mutation could obviously promote generation and accumulation of Aβ oligomers in an early stage. CTX, cerebral cortex; CA3, hippocampal CA3 region. Note that (F–J) and (P–T) are higher magnifications of (A–E) and (K–O), respectively. Scale bar represents 100 µm. (TIF) [file pone.0085885.s004.tif]

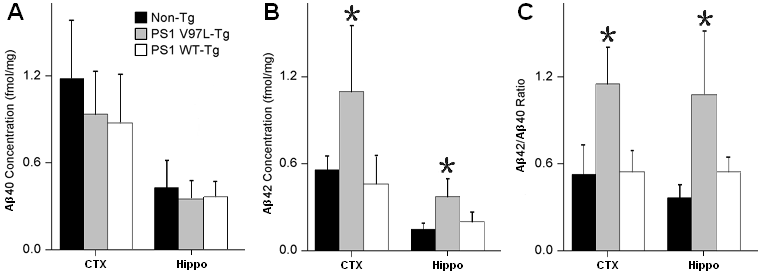

Supplement: Figure S5 — No change in the levels of Aβ40 and Aβ42 in PS1WT-Tg mice at 9 months. (A) Aβ40 expression level. (B) Aβ42 expression levels. (C) The ratio of Aβ42/Aβ40. ELISA measurements are from the cortex and the hippocampus of 9-month-old mice. * denotes a significant difference at p<0.05 vs Non-Tg littermates (n = 6/group). (TIF) [file pone.0085885.s005.tif]

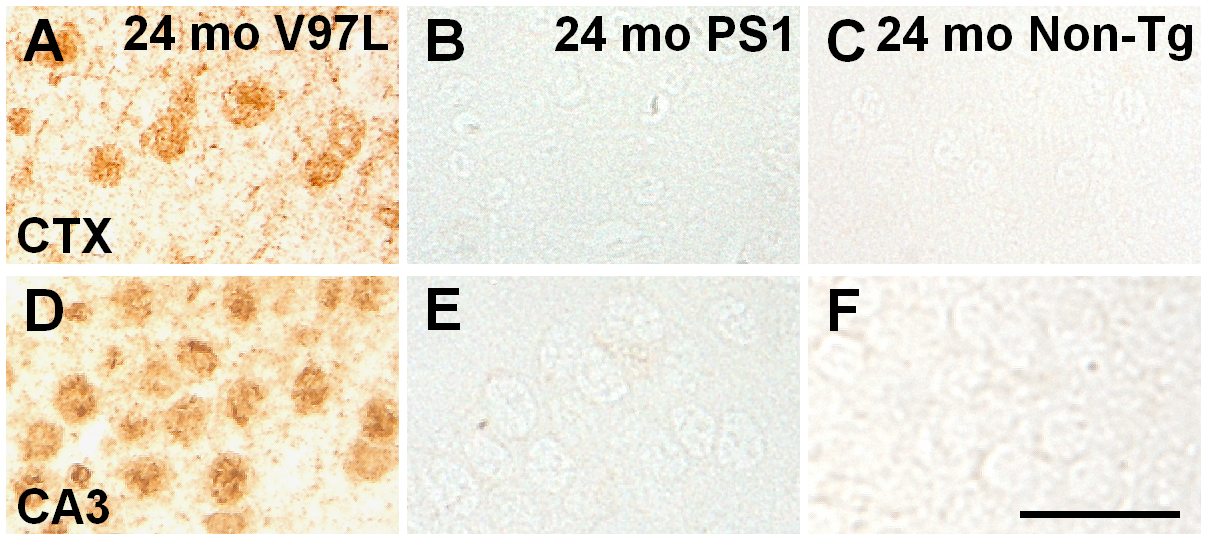

Supplement: Figure S6 — No positive abnormal tau hyperphosphorylation stain in PS1WT-Tg mice even at 24 months. (A–F) Detailed graphs of the cortex and hippocampus that show no intracellular accumulation of AT-8 staining in PS1WT-Tg mice, compared with positive control PS1V97L-Tg mice. Scale bar represents 50 µm. (TIF) [file pone.0085885.s006.tif]
